# Inflammatory Bowel Disease Care Barriers and Medication Beliefs in a Majority Hispanic Population: A Patient Survey

**DOI:** 10.1093/crocol/otaf058

**Published:** 2025-10-06

**Authors:** Florence-Damilola Odufalu, Aarti A Patel, Kathleen L Deering, Christian F Arias, Margaret Yung, Alicia C Shillington

**Affiliations:** Keck School of Medicine of University of Southern California, Los Angeles, CA, United States; Johnson & Johnson Innovative Medicine, Titusville, NJ, United States; EPI-Q Inc., Chicago, IL, United States; Keck School of Medicine of University of Southern California, Los Angeles, CA, United States; EPI-Q Inc., Chicago, IL, United States; EPI-Q Inc., Chicago, IL, United States

**Keywords:** inflammatory bowel disease, health care disparities, medication beliefs, social determinants of health, Hispanic populations

## Abstract

**Background:**

Data regarding barriers to inflammatory bowel disease (IBD) care in largely Hispanic populations is limited. This study evaluated barriers in Black/Indigenous/People of Color/Hispanics (BIPOC/H) and White/non-Hispanics (W/NH) with IBD.

**Methods:**

An anonymous survey was administered to adult IBD patients at Keck Medical Center of the University of Southern California and Los Angeles General Medical Center between March and October 2024. Data included the Consumer Assessment of Healthcare Providers and Systems and Barriers to Care surveys, the Medication Adherence Rating Scale-4, and the Beliefs about Medicines Questionnaire. Analyses compared BIPOC/H and W/NH patients via *X*^2^ and *t*-tests.

**Results:**

Included were 172 of 195 eligible patients (88% response). Compared to W/NH patients, BIPOC/Hs reported delays of longer than 1 year in receiving a diagnosis, 49.6% versus 27.8% (*P* = 0.015) more IBD-related financial difficulty, 55.6% versus 22.2% (*P* = 0.001); more difficulty obtaining emotional support (56.4% vs. 33.3%, *P* = 0.05); and community support for IBD (45.0% vs. 20.0%, *P* = 0.004). BIPOC/H patients had higher mean worries about medication harm (13.7 vs. 11.6, *P* < 0.001), concerns about long-term medication use (17.2 vs. 13.9, *P* < 0.001), and worries medications are overused (9.0 vs. 7.6, *P* < 0.002). Specific beliefs, including “medications are toxic” and concerns about dependency, were significantly more prevalent in BIPOC/H respondents (*P* < 0.05).

**Conclusions:**

BIPOC/H patients with IBD had more delays in diagnosis, medication-related concerns, IBD-related financial difficulties, and less social/emotional support for their IBD versus W/NHs, underscoring the need for culturally sensitive care, identification and communication of emotional, and community support resources, as well as medication decision support.

## Introduction

Inflammatory bowel diseases (IBDs) include Crohn’s disease (CD) and ulcerative colitis (UC), and are chronic, painful, recurring inflammatory conditions of the digestive system that affect quality of life.^1–3^ Early epidemiological studies of IBD largely concluded that it was a disease predominantly affecting non–Hispanic White populations, with lower rates observed in other racial and ethnic groups.^4–6^ However, a growing body of research has revealed an increased prevalence of IBD in people of color, including Hispanic, Black, and Asian populations. These findings challenge earlier assumptions and highlight the need for a deeper understanding of the unique challenges these populations face and the implications for the care team.^7–10^

In the limited studies focusing on IBD in historically marginalized communities in the United States, patients of color tend to be underinsured, have low household income, and are more likely to experience moderate-to-severe disability, regardless of disease severity.^11^ These disparities underscore the importance of targeted research and interventions to address the unique challenges faced by these populations. Delays in obtaining medical care have been observed in Hispanic patients and other populations of color, potentially contributing to more disease activity, more severe disease, and worse clinical outcomes.^12–14^ Disparities by race have also been observed in access to IBD specialty care and greater healthcare resource utilization, including emergency department visits.^15–17^ There is a paucity of data examining the role of race and ethnicity on attitudes and beliefs towards IBD medication use in Hispanics. However, data in Hispanic patients in other disease states, and in other populations of color suggest differences in medication attitudes and trust in clinicians that could impact medication acceptance and decision-making.^14^^,^^18^^,^^19^

This study was conducted to evaluate barriers to care, clinical disease status and outcomes among a diverse population of IBD patients treated at Keck Medical Center of University of Southern California (USC) and Los Angeles General Medical Center (LAGMC).

## Materials and Methods

This study aimed to examine patient-reported barriers to care, IBD symptoms, medication attitudes, access and adherence among a diverse population of patients with IBD treated at USC and LAGMC. Included patients were 18 years or older, with a diagnosis of either CD (ICD-10 K50.90) or UC (ICD-10 K51.x) prior to June 1, 2022.

The survey was adapted in English and Spanish with translations provided by De Miranda Medical based on a previous study conducted in a largely Black population.^20^ It was designed to incorporate social determinants of health (SDOH) domains, IBD symptoms, severity, access to care (including gastroenterology specialists and medications), a health literacy screener, medication adherence, and attitudes toward medication. The survey was available in online and paper format. Participants were identified from electronic medical records (EMR). Patients were screened and enrolled at their gastroenterology in-person clinic visit at either USC or LAGMC. Responses to the survey were anonymous, and patients were compensated with a $50 gift card for participating. This study was approved by the USC and LAGMC institutional review board (protocol #HS-23-00681).

Data collected in the survey included demographics, IBD history, and symptoms over the 7 days prior to the date the survey was completed. Access to IBD-related health care was assessed via an instrument incorporating consumer opinions of healthcare and barriers to care^20–22^ to understand geographical, psychosocial, and resource-related barriers to health care received in the 12 months prior to the date the survey was completed. Questions were administered on a 5-point Likert scale related to problems accessing care ranging from no problem (0) to a major problem (4).

The survey also included the Medication Adherence Rating Scale-4 (MARS-4)^23^^,^^24^ to measure medication adherence. The MARS-4 consists of a 5-point Likert scale that measures respondents’ agreement with statements about their medication-taking behavior. Scores range from always (1) to never (5). The individual item scores are summed to give a total score ranging from 4 to 20, with scores below 16 indicating lower adherence.

The Beliefs about Medicines Questionnaire (BMQ)^25^ assessed patients’ attitudes toward medications. This 18-item instrument evaluates beliefs across 4 subscales including medication necessity, concern about medications, overuse, and potential harm. Each item is scored on a 5-point Likert scale, ranging from strongly disagree (1) to strongly agree (5).

Finally, health literacy was assessed via the single item literacy screener (SILS), a 1-question instrument that asks patients to rate, on a 5-point scale (never to always), how often they need for help reading information received from a doctor or pharmacy. Scores of 2 or higher (sometimes) indicates lower health literacy.^26^

The analysis described and compared survey responses from Black, Indigenous, People of Color, and Hispanic (BIPOC/H) and White, non-Hispanic (W/NH) patients using bivariate methods. Clinical characteristics were analyzed, with continuous variables summarized by the mean, and standard deviation, while categorical variables were presented as counts and percentages for each category. Comparisons between W/NH and BIPOC/H and W/NH groups were conducted using chi-square tests or Fisher’s exact tests for cell counts below 5. Student *t*-tests were employed to compare group means for continuous variables.

## Results

### Patient characteristics

**Figure 1.**
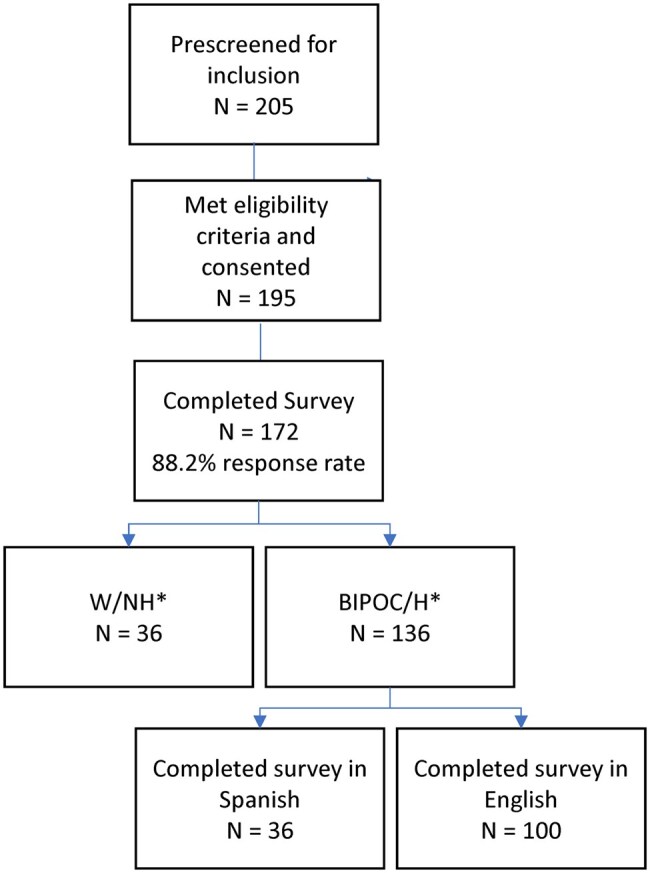
Patient disposition. *One English speaking patient and 2 Spanish-speaking patients opted to take the survey on paper.

Out of 205 pre–screened patients identified in EMR, 195 were eligible and offered participation at a clinic visit. Of these, 172 provided informed consent, enrolled, and completed the survey (88.2% response rate). Hispanic patients comprised 57% of the population, 9.3% identified as Black, 8.7% as Asian, and 7.6% as multi–racial (Figure 1). W/NH comprised 20.9% of the total population. Of the BIPOC/H patients, 36.0% completed the survey in Spanish. Mean age was 41 years in the BIPOC/H group versus 45 among W/NH patients (*P* = 0.146) with 39.7% and 55.6% being male, respectively (*P* = 0.093). Significantly fewer BIPOC/H patients had less than a high school education, 22.1% versus 0%, respectively (*P* < 0.001) (*P* ≤ 0.001). Private insurance coverage was significantly higher among W/NHs, 72.2% compared to the BIPOC/Hs, 32.4% (*P* ≤ 0.001). More W/NHs reported full-time employment: 52.8% versus the BIPOC/H group, 41.2% (*P* = 0.004). A significantly higher percentage of the W/NH group reported incomes over $100 000, 28.6% compared to 9.1% in the BIPOC/H group, (*P* = 0.001). The BIPOC/H group had a significantly higher proportion of individuals with literacy challenges (37.9%) compared to 16.7% in the W/NH group, *P* = 0.017. Substantially more BIPOC/H patients were underinsured, with only 32.4% versus 72.2% (*P* < 0.001) being privately insured (Table 1).

**Table 1.** Sociodemographics.

	**Total (*N*** **=** **172)**	**BIPOC/H (*n*** **=** **136)**	**W/NH (*n*** **=** **36)**	*P*
**Age**				
** Mean (SD)**	42 (14)	41 (13)	45 (17)	0.146
**Sex, *n* (%)**				
Male	74 (43)	54 (39.7)	20 (55.6)	0.093
Female	98 (57.0)	82 (60.3)	16 (44.4)	
**Race, *n* (%)**				
Black or African American	16 (9.3)	16 (11.8)	-	
Asian	15 (8.7)	15 (11.0)	-	
Other/multiracial	13 (7.6)	13 (9.6)	-	
**Ethnicity, *n* (%)**				
Hispanic	98 (57.0)	98 (72.1)	-	
Non-Hispanic	74 (43.0)	-	36 (100)	
**Education, *n* (%)**				
High school graduate	30 (17.4)	30 (22.1)	0 (0.0)	**<0.001**
**Type(s) of health insurance, *n* (%)**				
** Health Maintenace Organization (**HMO)/ Preferred Provider Organization (PPO)/private insurance^a^	70 (40.7)	44 (32.4)	26 (72.2)	**<0.001**
Medicare	52 (30.2)	44 (32.4)	8 (22.2)	0.309
Medicaid^a^	35 (20.3)	31 (22.8)	4 (11.1)	0.163
No insurance/self-pay	1 (0.6)	1 (0.7)	0 (0.0)	1.000
Other (eg, MediCal)	29 (16.9)	27 (19.9)	2 (5.6)	**0.046**
**Employment status, *n* (%)**				
Full time	75 (43.6)	56 (41.2)	19 (52.8)	**0.004**
Unemployed	48 (27.9)	45 (33.1)	3 (8.3)	
Part time/seasonal	26 (15.1)	22 (16.2)	4 (11.1)	
Retired^a^	8 (4.7)	3 (2.2)	5 (13.9)	
On disability	7 (4.1)	5 (3.7)	2 (5.6)	
Something else	8 (4.7)	5 (3.7)	3 (8.3)	
Household income, *n* (%)*				
<$20 000	24 (14.4)	20 (15.2)	4 (11.4)	**0.001**
$20 001-$40 000	21 (12.6)	16 (12.1)	5 (14.3)	
$40 001-$60 000	11 (6.6)	9 (6.8)	2 (5.7)	
$60 001-$80 000	4 (2.4)	3 (2.3)	1 (2.9)	
$80 001-$100 000*	10 (6.0)	4 (3.0)	6 (17.1)	
>$100 000^a^	22 (13.2)	12 (9.1)	10 (28.6)	
Declined to answer but note have faced financial difficulties due to IBD	33 (19.8)	29 (22.0)	4 (11.4)	
Prefer not to answer^a^	42 (25.1)	39 (29.5)	3 (8.6)	
**Single item literacy screener (SILS)** **≥** **2** ^a^	56 (33.3)	50 (37.9)	6 (16.7)	**0.017**

Bold p values indicate statistically significant at the .05 level.

*N* may not equal 172 as patients may have declined to respond to individual questions, and some questions allowed for multiple responses.

a Denotes a category whose column proportions differ significantly from each other at the 0.05 level in post hoc analysis.

Abbreviations: BIPOC/H, Black/Indigenous/People of Color/Hispanics; IBD, inflammatory bowel disease; W/NH, White/non-Hispanics.

### Care barriers, diagnostic delays, and severity

In terms of disease severity and history, W/NH patients were diagnosed on average 4 years earlier than BIPOC/H patients (*P* = 0.028). Nearly twice as many BIPOC/H patient reported having symptoms for longer than 1 year prior to receiving a diagnosis, 49.6% versus 27.8% (*P* = 0.015). More BIPOC/H patients reported symptoms being poorly or very poorly controlled than W/NHs 26.1% versus 16.7%, however, likely due to inadequate sample size, this was not statistically significant. Regardless of race or ethnicity, 28.5% had ever had an IBD-related surgery, 34.3% reported a hospital admission in the 12 months prior to the survey date, and 32.6% required an emergency department visit (Table 2).

**Table 2.** IBD symptom history and severity.

	**Total (*N*** **=** **172)**	**BIPOC/H** (***n*** **=** **136)**	**W/NH (*n*** **=** **36)**	*P*
**Disease duration**				
** Mean (SD)**	11 (9)	10 (9)	14 (13)	**0.028**
**Length between symptom onset and diagnosis, *n* (%)**				
** Less than 1 year**	94 (55.0)	68 (50.4)	26 (72.2)	**0.015**
**One year or longer**	77 (45.0)	67 (49.6)	10 (27.8)	
**Patient-reported current level of symptom control, *n* (%)**				
**Well/fairly well controlled**	129 (75.9)	99 (73.9)	30 (83.3)	0.279
**Not well/poorly/very poorly controlled**	41 (24.1)	35 (26.1)	6 (16.7)	
**Hospital admission for IBD in the last 12 months**	59 (34.3)	50 (36.8)	9 (25.0)	0.417
**ER or urgent care visit for IBD in the last 12 months**	56 (32.6)	47 (34.6)	9 (25.0)	0.396
**Ever had surgery due to IBD**	49 (28.5)	38 (27.9)	11 (30.6)	0.836

Bold p values indicate statistically significant at the .05 level.

*N* may not equal 172 as patients may have declined to respond to individual questions.

Abbreviations: BIPOC/H, Black/Indigenous/People of Color/Hispanics; IBD, inflammatory bowel disease; W/NH, White/non-Hispanics.

### Financial and disease support impacts

**Figure 2.**
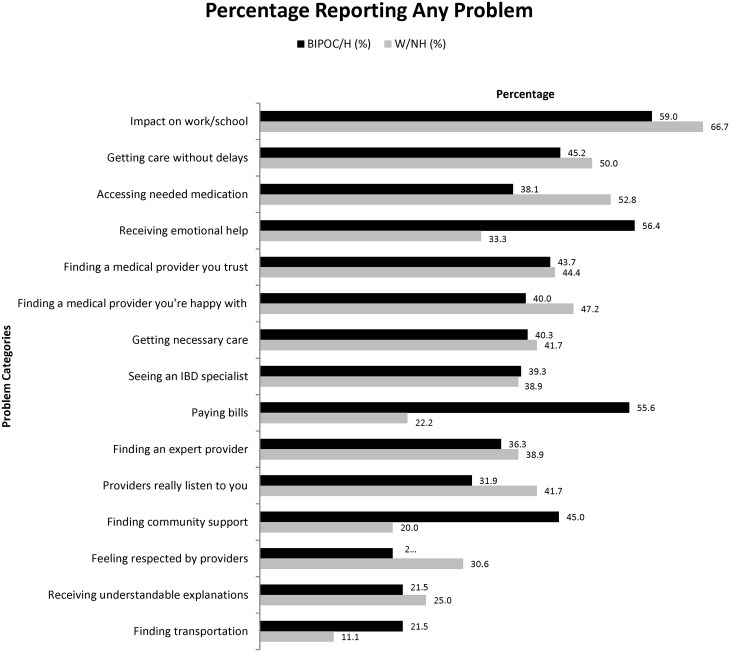
Barriers to care—patients reporting any problems. *N* may not equal 172 as patients may have declined to respond to individual questions.

When asked about specific impacts and barriers due to IBD in this population, BIPOC/H patients were significantly more likely than W/NH to report difficulties with paying bills due to IBD (55.6% vs. 22.2%, *P* < 0.001), receive emotional help for IBD (56.4% vs. 33.3%, *P* = 0.05), and finding community support for IBD (45.0% vs. 20.0%, *P* = 0.004), respectively. Regardless of race or ethnicity, between 30% and 40% or greater of all patients reported challenges in obtaining necessary care without delays, finding medical providers they trusted, being satisfied with their care and feeling heard by their providers. Nearly 40% also reported difficulty accessing needed medications. Approximately 60% of all patients reported problems working or going to school related to their IBD. These additional barriers were not statistically different between racial/ethnic demographics (Figure 2).

### Medication beliefs and adherence

With respect to medication attitudes as measured by the BMQ, BIPOC/H patients more often endorsed the belief that their IBD medications were necessary, versus W/NH patients. However, BIPOC/H patients reported higher mean (SD) scores on the medication Harm subscale 13.7 (3.3) versus 11.6 (3.0), (*P* < 0.001), including expressing more worry about medications being toxic or addictive versus W/NHs. They also reported significantly higher mean (SD) scores on the medication concern subscale, 17.2 (4.0) versus 13.9 (3.8), (*P* < 0.001), including worries about long-term effects of medications, becoming dependent upon medication, and the disruption medication had on their lives. BIPOC/H patients also reported higher mean (SD) scores on the medication overuse subscale 9.0 (2.2) versus 7.6 (2.6), including more concerns that doctors put too much trust in medications, and if doctors had more time to spend with patients, they would use less medication. The additional concern about medications and fear of harm, did not translate into statistically significant medication adherence differences in BIPOC/H patients via the MARS-4 (Table 3).

**Table 3.** Beliefs about medicines and non–adherence.

	**Total (*N*** **=** **172)**	**BIPOC/H (*n*** **=** **136)**	**W/NH (*n*** **=** **36)**	*P*
		Mean (SD)	Mean (SD)	
**Medication necessity subscale score**	18.7 (3.9)	19.2 (3.9)	17.3 (3.6)	**0.01**
** Currently my health depends on my medication**	4.1 (1.0)	4.2 (1.0)	4.0 (1.2)	0.538
** My life would be impossible without my medication**	3.8 (1.3)	3.8 (1.2)	3.6 (1.3)	0.247
** Without my medication I would be very ill**	4.1 (1.0)	4.2 (1.0)	3.8 (1.1)	**0.038**
** My health in the future depends on my medication**	4.0 (1.0)	4.1 (1.0)	3.9 (1.1)	0.315
** My medication prevents my condition from getting worse**	4.2 (0.9)	4.3 (0.8)	4.0 (0.9)	0.081
**Medication harm subscale score**	13.2 (3.3)	13.7 (3.3)	11.6 (3.0)	**<0.001**
**People who take medication should stop for a period of time once in a while**	2.4 (1.1)	2.5 (1.1)	2.1 (1.1)	0.093
** Most medications are addictive**	2.5 (1.1)	2.6 (1.1)	2.0 (0.9)	**<0.001**
** Natural remedies are safer than medicines**	2.8 (1.1)	2.9 (1.1)	2.5 (1.1)	0.063
** Medicines do more harm than good**	3.0 (0.9)	3.0 (0.9)	3.1 (1.1)	0.734
** All medications are toxic**	2.5 (1.1)	2.7 (1.1)	1.9 (1.0)	**<0.001**
**Medication concern subscale score**	16.5 (4.2)	17.2 (4.0)	13.9 (3.8)	**<0.001**
** Having to take medications worries me**	3.3 (1.2)	4.1 (1.0)	3.8 (1.1)	0.079
**I sometimes worry about the long term effects of my medication**	4.0 (1.0)	4.2 (1.0)	3.6 (1.2)	**0.008**
** My medications are a mystery to me**	2.9 (1.1)	2.9 (1.1)	2.8 (1.1)	0.220
** My medication disrupts my life**	2.7 (1.2)	2.9 (1.2)	2.0 (1.0)	**<0.001**
**I sometimes worry about being too dependent on my medication**	3.5 (1.3)	3.8 (1.3)	2.7 (1.3)	**<0.001**
**Medication overuse subscale score**	8.7 (2.4)	9.0 (2.2)	7.6 (2.6)	**<0.002**
** Doctors prescribe too many medications**	2.8 (0.9)	2.9 (0.9)	2.8 (1.1)	0.478
** Doctors put too much trust in medications**	3.0 (1.2)	3.2 (1.2)	2.4 (1.1)	**<0.001**
**If doctors spent more time with patients they would prescribe few medications**	2.8 (0.9)	2.9 (0.9)	2.4 (1.1)	0.**024**
**Non–adherent, *n* (%)** ^a^	40 (23.8)	35 (26.1)	5 (14.7)	0.184

Bold p values indicate statistically significant at the .05 level.

*N* may not equal 172 as patients may have declined to respond to individual questions.

Abbreviations: BIPOC/H, Black/Indigenous/People of Color/Hispanics; W/NH, White/non-Hispanics.

a MARS score < 16.

## Discussion

In a largely Hispanic population treated in gastroenterology clinics at USC and LAGMC, this study found substantial barriers to patients obtaining an IBD diagnosis, accessing care, concerns regarding the long-term use of medications to treat IBD, inadequate emotional support, and negative financial impacts due to their condition.*Notably, irrespective of race and ethnicity, a large proportion of patients reported they had difficulty in obtaining the care they need, a recognized risk factor for negative IBD-related outcomes.^27^^,^^28^ Nearly half of all patients studied reported difficulty accessing healthcare providers and essential IBD care without delays. BIPOC/H patients reported significantly longer lengths of time between symptom onset and receipt of a diagnosis, with nearly half of BIPOC/H patients having diagnostic delays of longer than 1 year.

Delayed diagnosis of IBD has been associated with adverse long-term outcomes, including increased risks of intestinal surgery, higher health resource utilization, and disease complications. In this study, BIPOC/H patients reported symptoms were not well controlled in more than a quarter of the population. Jayasooriya et al. demonstrated in a 2023 meta-analysis that in high income countries such as the United States, the median time to diagnosis was 6.2 months for CD and 3.2 months for UC, with diagnostic delays impacting severity and clinical outcomes, including increasing the odds of stricturing CD (odds ratio [OR] = 1.88; confidence interval [CI]: 1.35-2.62), penetrating CD (OR = 1.64; CI: 1.21-2.20), and IBD-related surgery (OR = 2.24; CI: 1.57-3.19).^29^ Likewise, Nguyen et al.^30^ and Schoepfer et al.^31^ found increases in overall complications as high as 8-fold, particularly intestinal strictures and stenosis, with longer diagnostic delays. In an era where broader societal emphasis is shifting away from reducing racial and ethnic disparities, this study underscores a continuing and substantial unmet need of Hispanics in obtaining effective care expeditiously, increasing their likelihood of complications with related health and cost consequences.

BIPOC/H patients in this study reported significantly more challenges in SDOH compared to W/NHs, including more financial strain due to IBD, higher rates of underinsurance, lower health literacy, and educational attainment. Given the broader IBD delays in diagnosis and symptom duration differences prior to obtaining treatment between BIPOC/H and W/NH patient groups, these factors need to be considered when providing care to this population. Damas et al. similarly found that 56% of Hispanic IBD patients experience financial strain, a higher percentage than their non-Hispanic Black (40%) and non-Hispanic White (25%) counterparts.^12^ This financial burden may contribute to delayed medical care, with approximately 30% of patients postponing treatment due to costs, further exacerbating disease severity and increasing hospitalization rates.^12^ Burbage et al. and Shah reported that BIPOC/H patients incur greater direct medical expenses and more frequent healthcare visits, contributing to high financial burden.^20^^,^^32^ These disparities illustrate the need for targeted interventions to alleviate financial obstacles and improve access to care for Hispanic individuals suffering from IBD.^15^

BIPOC/H individuals in this study were significantly more likely to report challenges in accessing support for coping with the emotional impacts of their IBD. Social isolation, insufficient interaction and engagement, and a lack of social support have been linked to higher burden of gastrointestinal symptoms in people living with IBD. The unpredictable nature of IBD, emotional aspects of managing symptoms, and difficulties in maintaining daily activities and employment are well-documented challenges for these individuals.^33^ Patients in this study highlighted the inability to access social and emotional support for their IBD. Shah et al. similarly found a largely Black population of patients with IBD reported significantly more difficulty accessing and receiving emotional support to deal with IBD (18% vs. 42%) and that they had inadequate access to community support to deal with IBD (29% vs. 17%) versus W/NH patients, respectively.^20^ These reinforces the need for integrated mental health support in IBD care models, ensuring that psychosocial aspects of the disease are addressed alongside medical management.

Misconceptions about medication use are well-documented in patients of color. In our study, notable patient attitudes that interfere with the acceptance of medications, included worries about becoming dependent on IBD medications, fears related to addictiveness and toxicity, and worries about the impact of long-term use. In other disease states, Huang reported similar findings in type 2 diabetes, with BIPOC/H patients expressing more concerns than W/NHs regarding drug side effects (66% and 49% vs. 39%) and medication dependency (65% and 52% vs. 39%), respectively.^34^ BIPOC/H patients had more significant limitations in health literacy based upon the SILS, which may impact difficulties in medication acceptance and navigating the complexities of IBD management, important considerations when guiding patients through successful treatment.

### Limitations

This study is subject to several limitations. The patients surveyed were those receiving care for IBD at a USC or LAGMC gastroenterology practice and thus includes sampling bias and may represent a more advanced or refractory IBD population given their care is primarily at a tertiary care center or safety-net hospital. As patients were in treatment with a gastroenterologist that specialized in IBD, our findings may not fully capture the experiences of patients who remain undiagnosed or those who seek care in primary care settings. Thus, the study likely underestimates barriers faced in a broader Hispanic IBD community without sufficient access to specialty care. Barriers to care would likely be substantially greater if the survey included patients unable to receive care from a gastroenterologist. Although the survey was anonymous, stigma or reluctance to discuss digestive issues might lead to underreporting of symptoms. Variability in health literacy can also impact the understanding of medical terms related to IBD. Respondents with limited knowledge may struggle to accurately describe their experiences or symptoms, leading to incomplete or inaccurate data. Study results are generalizable only to patients with characteristics similar to those included in this study during the study time frame. Data may not be generalizable to other populations or similar populations treated in different time frames, dependent upon prior, current, and emerging standards of care. As we did not have information on non–survey respondents, we were not able to determine differences between survey respondents and non–responders. Data are primarily patient-reported and are subject to recall bias.

## Conclusions

This is a comprehensive study of sociodemographics and barriers to care in a largely Hispanic patient population with IBD. The study underscores the need for targeted support to address socioeconomic, cultural, and healthcare system barriers in IBD management, and social and emotional support for people living with IBD. Both BIPOC/H and W/NH patient demographic groups face challenges is accessing care. While BIPOC/H patients place high importance on the role of medication in controlling their IBD, they also have significant concerns about medication safety. Addressing these concerns through culturally tailored interventions, improved patient-provider communication, increasing under-represented populations in policy and clinical research may enhance medication adherence and long-term disease management. Addressing financial, educational, and cultural factors in healthcare delivery, particularly in underserved populations, may improve health outcomes and quality of life for IBD patients across racial and ethnic backgrounds. Future efforts should focus on community-based interventions and policy initiatives that promote health equity in IBD care.

## Data Availability

Data are available upon request.
